# Exploring motion using geometric morphometrics in microscopic aquatic invertebrates: ‘modes’ and movement patterns during feeding in a bdelloid rotifer model species

**DOI:** 10.1186/s40462-024-00491-9

**Published:** 2024-07-13

**Authors:** Andrea Cardini, Giulio Melone, Paul O’Higgins, Diego Fontaneto

**Affiliations:** 1 Dipartimento di Scienze Chimiche e Geologiche, Università di Modena e Reggio Emilia, Via Campi 103, 41125 Modena, Italy; 2 School of Anatomy, Physiology and Human Biology, The University of Western Australia, 35 Stirling Highway, Crawley, WA 6009 Australia; 3 Università degli Studi di Milano, 20100 Milan, Italy; 4 Department of Archaeology and Hull York Medical School, University of York, York, YO10 5DD UK; 5 Consiglio Nazionale Delle Ricerche (CNR), Istituto di Ricerca Sulle Acque (IRSA), Corso Tonolli 50, 28922 Verbania Pallanza, Italy; 6 National Biodiversity Future Center (NBFC), Piazza Marina 61, 90133 Palermo, Italy

**Keywords:** Behaviour, Procrustes shape, Motion analysis

## Abstract

**Background:**

Movement is a defining aspect of animals, but it is rarely studied using quantitative methods in microscopic invertebrates. Bdelloid rotifers are a cosmopolitan class of aquatic invertebrates of great scientific interest because of their ability to survive in very harsh environment and also because they represent a rare example of an ancient lineage that only includes asexually reproducing species. In this class, *Adineta ricciae* has become a model species as it is unusually easy to culture. Yet, relatively little is known of its ethology and almost nothing on how it behaves during feeding.

**Methods:**

To explore feeding behaviour in *A. ricciae*, as well as to provide an example of application of computational ethology in a microscopic invertebrate, we apply Procrustes motion analysis in combination with ordination and clustering methods to a laboratory bred sample of individuals recorded during feeding.

**Results:**

We demonstrate that movement during feeding can be accurately described in a simple two-dimensional shape space with three main ‘modes’ of motion. Foot telescoping, with the body kept straight, is the most frequent ‘mode’, but it is accompanied by periodic rotations of the foot together with bending while the foot is mostly retracted.

**Conclusions:**

Procrustes motion analysis is a relatively simple but effective tool for describing motion during feeding in *A. ricciae*. The application of this method generates quantitative data that could be analysed in relation to genetic and ecological differences in a variety of experimental settings. The study provides an example that is easy to replicate in other invertebrates, including other microscopic animals whose behavioural ecology is often poorly known.

## Introduction

An important component of behaviour is the “sequence of movements performed by an animal… But which movements should we consider? And how should we group them together?” (p. 218 [1]). The quantitative analysis of animal motion is a type of computational ethology [2] that seeks to provide an accurate numerical description of movement in space and time. Using a variety of approaches, researchers can track the position of one or more individuals and describe movement in terms of changes in body posture [2, 3]. It is this second objective that is the main focus of our study, where we measure the variation in the relative positions of body parts during motion in a microscopic aquatic invertebrate. More precisely, using video recordings of the freshwater meiobenthic bdelloid rotifer *Adineta ricciae* Segers & Shiel 2005, we assess the occurrence of an underlying low dimensional simplicity^1^ [4] in its movement and explore whether postures can be grouped into ‘modes’ of similar stereotyped motions [1, 5]. By stereotyped motions we mean well-defined behavioural acts, which are repeated and can, to some extent, be separated into elements [6]. However, as we explain in more detail below, we specifically focus on changes in posture while searching for food and feeding, which happens after an animal stops in a given spot, where, presumably, it can find its microscopic food, such as organic detritus, bacteria, algae, protozoans and tiny invertebrates [7]. Accurately measuring an aspect of animal behaviour in meiobenthic organisms has the potential to improve our knowledge in lineages rarely considered by ethologists. It also helps reducing subjectivity in the detection of subtle behavioural details [2], while generating numerical data for comparative studies and experimental research.

*Adineta ricciae* is a model organism for ecological and evolutionary research, because of its ability to thrive under laboratory conditions [8–10]. Species of the genus *Adineta*, such as *A. ricciae*, and the more common and widespread but morphologically very similar and closely related *A. vaga* (Davis, 1873), can be found in temporary water bodies and/or in limno-terrestrial habitats such as soils, mosses, and lichens [11]. All the species of the genus move actively on a surface using both ciliary beating of the flat antero-ventral corona of cilia and contractions of their foot, which can stick to the surface. These animals feed by scraping microorganisms off surfaces. When they do this, they keep the ciliated corona on the ventral side of the head moving back and forth and convey food to the mouth with a rake at the base of the ciliated corona as they continuously shorten and expand their body while leveraging on the foot [7]. After collecting the food in an area, they leave rapidly and likely move to a new feeding spot. When they move to a new spot to search for food and feed, it is almost impossible to keep recording their behaviour under a microscope, as the field of view, with the zoom required for an appropriate video resolution, is too narrow to track the departing animal.^2^ Thus, our behavioural analysis does not consider the movement during the displacement phase between two feeding spots and is restricted to the scraping of a surface for collecting food, that we call “feeding”.

For the quantification of feeding motion in *A. ricciae*, we apply a Procrustes motion analysis [12, 13]. This technique borrows the Procrustes superimposition [14, 15], routinely employed as a method for feature extraction in geometric morphometrics [16, 17], to minimise positional differences (translation, rotation, and also size) between video-recorded motion frames of a sample of individuals described by a common set of anatomical landmarks. With a Procrustes motion analysis, we can, thus, measure changes in postures (‘animal pose’) during feeding with an ‘egocentric’ representation, which is independent of position and orientation [2]. Size is standardised and separately considered to avoid that the large variation in body size in a soft bodied animal completely dominates the pattern of change. Compared to simpler analyses, such as tracking the movement of the centroid of an animal, a landmark-based morphometric approach also includes information on organismal form changes. Thus, the analysis captures the connection between feeding and body shape [4] in a species whose behaviour is almost completely unstudied, despite the simplicity with which it can be kept in laboratory conditions and its widespread use as a model organism [10, 18]. In addition to visualizing shape changes [19], Procrustes motion analysis allows the possibility of going back and forth between the statistical space of the numerical description of a moving organism and the physical representation of motion based on the landmark configuration.

In the study, we first employ Procrustes motion analysis to summarise the main patterns of motion during feeding in a sample of *A. ricciae* kept in the laboratory in standardised conditions. We, then, search for clusters (‘modes’) of relatively similar postures and use the clusters to interpret concomitant changes in position, orientation, and size of an individual. Using this information, we further explore whether splitting a complex, continuous motion into ‘modes’ improves our understanding of the variability in feeding behaviour of *A. ricciae*. Finally, we discuss both the limitations and future directions of our work. Besides specific information on the motion and biology of *Adineta*, our analysis offers an example that is easy to apply to other organisms to measure behavioural responses to ecological and environmental factors, as well as to experimental manipulation. The approach is also applicable to taxonomic comparisons and, more generally, to the analysis of phenotypic and functional differences in relation to genetic variability in nature and in the laboratory.

## Methods and materials

### Data and samples

Animals come from a culture that was kept under laboratory conditions with a continuous supply of food for years, after the original animals were collected in Australia and then became a model organism for different studies in bdelloid research [8–10]. Animals were kept under constant hydration, and day/night light conditions of 12/12 h [20]. Adult individuals were individually extracted from the culture, placed on a clean glass slide with a drop of water from the culture medium, kept under a coverslip, and observed using a compound microscope at 100× magnification. The space between the slide and the coverslip allowed the animals to move apparently in a natural way while searching for food on the slide. When an animal is compressed in a narrow space and cannot freely move, e.g. by compressing manually the coverslip, it tends to show the displacement phase and tries to move to a more convenient spot (personal observation). Each slide was cleaned before using it for observation; therefore, no food was available for the animals.

A digital camera (Nikon CMOS) mounted on the microscope (Nikon Eclipse 80i) recorded 2D videos of 51 individuals using Image Pro Plus. For each individual, we analysed only one video, the one with the longest time spent by the animal apparently scraping the slide for food and holding the foot on the slide, with the ventral side of the animal facing the slide and the dorsal side facing the coverslip. The zoom of the camera was held constant and, thus, the scaling factor to measure size on the digital images was always the same (1.386 micron/pixel). Videos were split into a series of frames, with each pair of consecutive frames separated by an identical time lag of 50 ms. This produced for the whole sample (i.e., all individuals and frames) a total of 3682 frames, which became 3501 after removing non-feeding frames (i.e., frames recording an individual moving away, after scraping the slide for food in a specific spot).

As the videorecording begins when an individual comes into view on the slide, the initial state of a feeding sequence, as well as its duration, which depends on how long it fed in that specific spot, varies across individuals in the sample. Some individuals moved from one feeding spot to another, in and out of the view of the camera. This means that for many individuals there are missing motion frames with video recording stopped and then restarted in a different spot. To better capture the range of postures during feeding, we included in the main analysis sample only individuals with a relatively large number of continuous frames (≥ 50) and focused on those capturing an uninterrupted feeding session in the same spot. This resulted in a subsample of 31 individuals with 54 to 100 frames each (i.e., feeding sessions lasting ~ 3–5 s). In this dataset, we further standardised the duration of feeding by selecting the first 54 frames for all individuals in the sample. Therefore, the main analysis sample (MAS) consisted of 31 individuals and a total of 1,674 frames. Having the same number of frames for each individual provides a dataset that is simpler to analyse and interpret, as time is kept constant. It also allows us to have the equivalent of a hold-out sample of 1827 frames for exploring the generalizability of results, as we briefly exemplify at the end of the Discussion. However, within the analytical framework we used, standardizing the duration of the behaviour being recorded is an optional step and using the total sample might be more powerful and accurate (see Discussion).

**Fig. 1:**
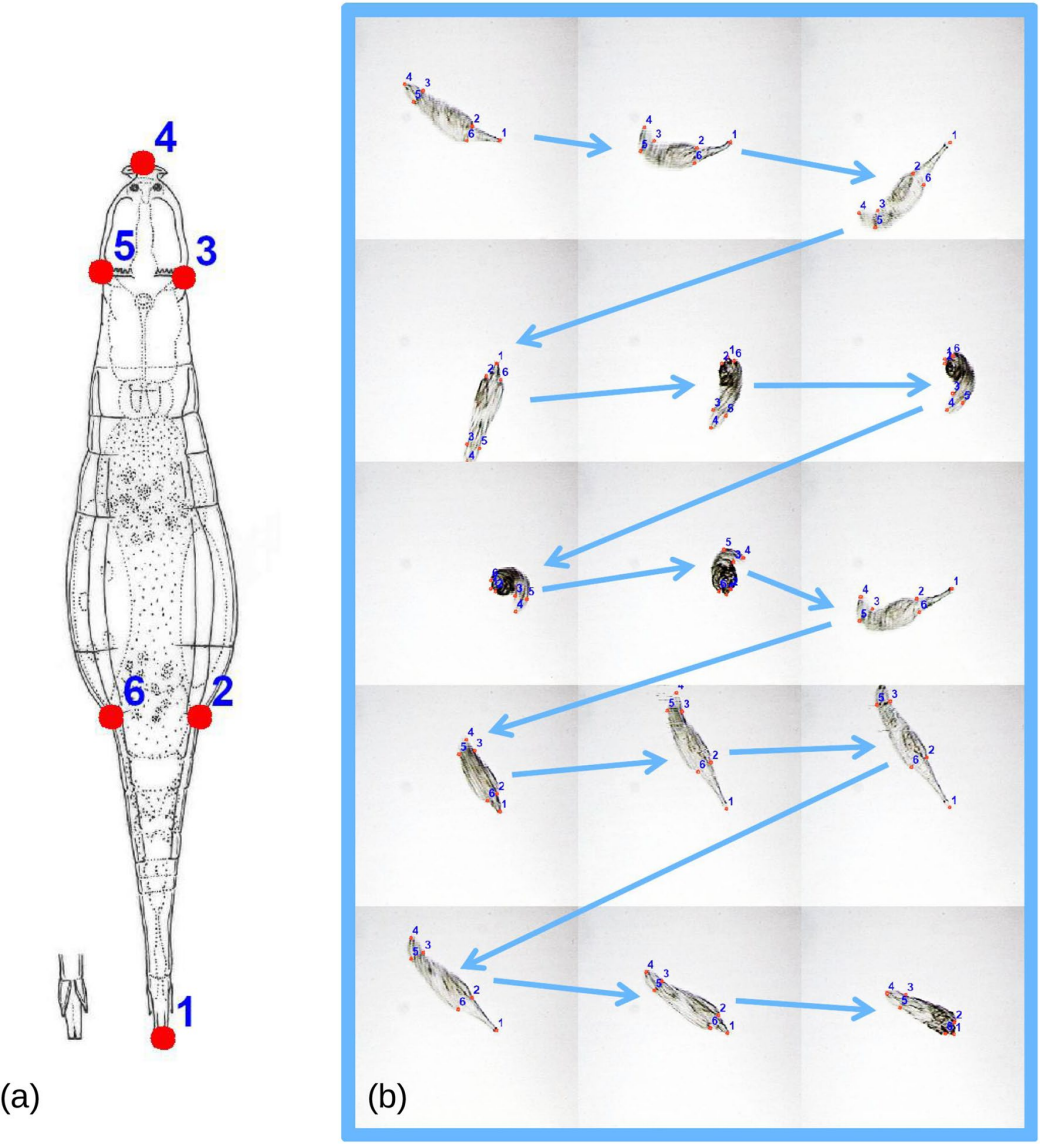
Configuration and example of data: **a** anatomical landmarks measuring body form (1, tip of the foot; 2, 6 base of the foot; 3, 5 neck; 4 tip of the head); **b** example of landmarked frames of an individual during feeding (arrows show consecutive frames)

To quantify changes in body form during feeding, the same operator (DF) digitised in TPSDig 2 [21] six anatomical landmarks on each individual frame (Fig. 1a). The landmark configuration was chosen so that these anatomically corresponding points parsimoniously capture how the size and shape of the main body regions (head, trunk and foot) change as an individual feeds. Shape is defined as the information left in a set of measurements once size is standardised and positional (i.e., translational and rotational) differences are minimised. The method used to extract size and shape is explained in the next subsection. As a convention, for simplicity, in the paper we use the term ‘frame’ to indicate each observation consisting of a set of variables corresponding to the six landmarks measuring body posture in that frame. Figure 1b presents a series of 15 landmarked frames in a specific individual. Because a frame is a 2D photograph of a three-dimensional organism, there is a potential distortion of, and a loss of information in, the missing third dimension [22]. However, *A. ricciae* is a relatively flat animal [23], which moves on surfaces in the film of water between particles of sediment or, in our experimental settings, between a slide and a coverslip under a microscope. For these reasons, as well as because the range of shape change is very large (see Results), the 2D approximation of this three-dimensional animal should be excellent. Also, digitization error (briefly mentioned here, for brevity, and assessed using duplicated digitization in one individual and the protocol of Viscosi & Cardini [24]) was negligible, with size and shape variation being respectively 309 and 49 times larger than average differences between duplicates.

### Feature extraction

**Fig. 2:**
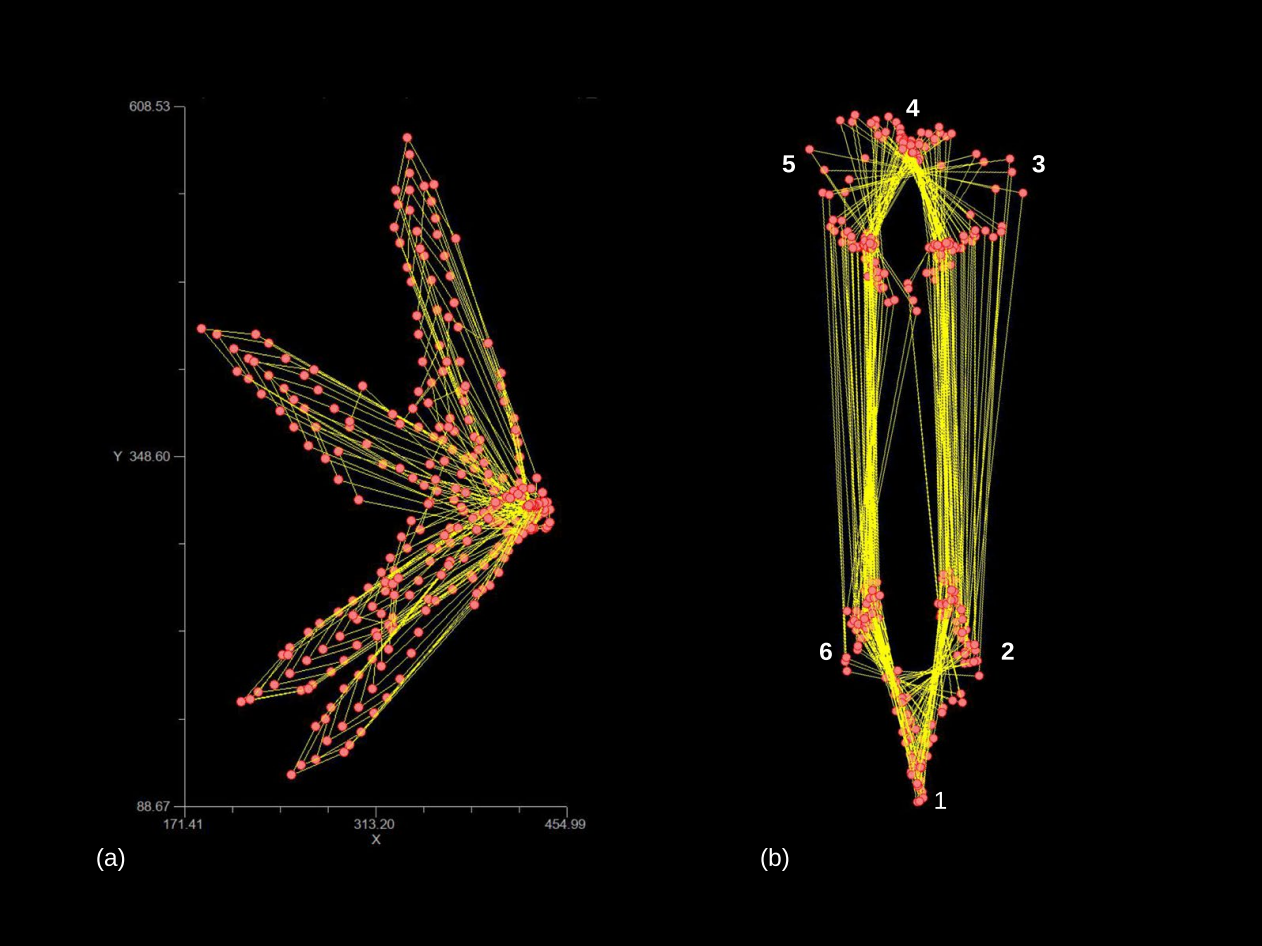
Individual 47 before (**a**) and after (**b**) the Procrustes superimposition (unit of measure is microns in (**a**) and not shown in (**b**), as, in this and other figures showing shape, units of Procrustes shape distance are arbitrary and specific to the configuration). In (**b**) landmark numbers of a single frame are shown to aid the interpretation

To measure body shape variation during feeding in *A. ricciae*, using a Procrustes superimposition [14, 15], we computed the Procrustes shape coordinates of the 12 raw coordinates (two, X and Y, for each landmark) of the matrix for the MAS of 31 individuals. Thus, size, estimated by the centroid size (CS) of the configuration^3^ of the landmarks in each frame (i.e., a measure of the dispersion of the landmarks around their ‘baricentre’), was standardised, frame by frame; configurations from all frames were centred in the centroid; rotational differences were minimised using a least squares method. In Fig. 2, we show, as an example, the landmark data of the 54 frames of one individual (identified as number 47) before (raw coordinates) and after (Procrustes shape coordinates) the superimposition.

The configurations after the superimposition lie on the surface of a curved shape space [25], which must be projected into a flat Euclidean space tangent to the sample mean shape. The projection is analogous to an orthogonal projection of the Earth surface on a flat map and is necessary because most statistical methods in biology require data to be in a Euclidean space, where the difference between two observations is measured by a straight line in the multivariate space. As in geographical maps of the Earth, the projection implies a degree of distortion that must be assessed. To explore the goodness of the tangent space approximation to the curved Procrustes shape space, Euclidean distances between each pair of the 1,674 observations were regressed through the origin onto the corresponding Procrustes shape distances.^4^ For the regression, we used TPSSmall [21]. If the approximation is good, as distances are nearly identical, the Pearson correlation (r) between the two types of distances should be virtually equal to one and the slope of the regression line should also be approximately one (i.e., 45°). This is what typically happens in biological data, whose shape variation occupies a small region of the total shape space (i.e., the space of all possible geometric shapes for a given landmark configuration) [26]. Thus, as the largest possible distance in the total shape space is π/2 = 1.57 [21], most distances should be much smaller than π/2 for a good approximation.

Depending on the analysis, the Procrustes superimposition was done either in the TPS Series [21] or in Morpho [27]. In both programs, we did not allow the algorithms to mirror reflect data to improve the fit, because distinguishing between turns to one or the opposite side is potentially useful information in a behavioural analysis. Specifically, we used the TPS Series for assessing the tangent space approximation in TPSSmall 1.36 and for the visualization of shape change in TPSRelw 1.75; however, we carried out all other analyses in R 4.3.1 [28], after superimposing the data using Morpho 2.11 [27].

Besides shape and CS, we computed another pair of variables, which may be useful to describe the feeding behaviour of *A. ricciae*. The first is the position of the centroid of the raw landmark configuration, which can be used to track an individual movement in space and is simply computed as the mean of the raw coordinates of the six landmarks in each frame. The second variable is the rotational angle of the foot, which is informative regarding the magnitudes and directions of rotation during feeding. The ‘foot angle’, as we called this variable for brevity, is computed using the vector connecting the tip of the foot (landmark 1) with the mid-point between the two landmarks at the base of the foot (landmarks 2 and 6). Alternatively, to quantify the rotation of the body in a feeding spot, we could have used the major axis of the landmark configuration instead of the foot angle. We opted for the foot angle, however, because *A. ricciae* feeds by attaching the toes at the tip of the foot to a specific spot for most or all the feeding session, which makes tracking the angular changes of the foot more interesting. Besides, if we used the long axis of the body, its estimated direction might be strongly affected by bending, which we have already measured in terms of body shape and that, in fact, as we show, dominates the variability in posture during feeding. Thus, to measure the foot angle during feeding in each individual sequence, we computed the angle in degrees between this vector at time t and the vector at time t-1, which corresponds to the direction of the foot in one frame relative to its direction in the previous frame. As a convention, we used positive angles for clockwise rotations and negative ones for anticlockwise rotations. Finally, we calculated the cumulative angle since the start of each motion sequence for each individual frame by summing the angles over previous frames (i.e., t + t + 1 + t + 2 + t + 3 etc.). Cumulative angles measure how much an individual has rotated overall in relation to its initial position. All the computations of raw centroids and foot angles were done in R. For the angles, we used an R function^5^ to compute the angle between two vectors in radians, which we later converted to sexagesimal degrees.

### Statistical analysis

#### Search for ‘modes’ of motion during feeding

In the first part of the study, we summarised and explored patterns in shape variation using a principal component analysis (PCA) of the variance covariance matrix of the MAS Procrustes shape coordinates. For the PCA, we used the R function *prcomp {stats}*. In the TPS Series, a PCA of Procrustes shape variables is called relative warp analysis [21]. Although computed differently [29], a relative warp analysis using the default options is equivalent to a conventional PCA. Thus, even when done to visualise shape variation in TPSRelw, we will adopt the conventional name ‘PCA’ to avoid redundant terminology. In a PCA of Procrustes shape variables, the dimensionality of the full data space is not the same as in the original matrix of landmark coordinates, because four degrees of freedom are removed during the 2D Procrustes superimposition: one when CS is standardised; another two because all data are translated along X and Y to be centered in the centroid; another one because of the least square minimization of rotational variation. This means that the PCA of motion data in *A. ricciae* produced only eight informative PCs with non-zero variance.

To explore whether Procrustes motion analysis suggests clusters of body shape postures that might represents ‘modes’ of motion, we used a k-means cluster analysis, as in Park et al. [30]. As is well explained by Pereira et al. (p. 1543[2]), the search for clusters classifies frames at “a given time point into a distinct … ‘state’ … [assuming] that data belonging to the same state exhibit similar, stereotyped dynamics given some measure of similarity”. In our case, the measure of similarity is the Euclidean shape distance computed pairwise between all frames. For the analysis, we used the function *kmeans {stats}* and set the number of clusters with the function *fviz_nbclust* of the R package *factoextra* [31]. The *fviz_nbclust* function repeats the k-means cluster analysis using different numbers of clusters (up to 20, in our case) and plots the average within cluster sum of squares (SSQ) in relation to the number of clusters. Similar to a scree-plot, the elbow of the plot suggests a parsimonious number of clusters, below which the reduction in within SSQ (and thus the separation of the clusters) levels off. For the *kmeans* function, the options we employed were three clusters (see Results) and 25 random sets to start the search^6^ with the Hartigan-Wong algorithm. The resulting clusters (‘modes’ of motion) were plotted as groups in the PCA scatterplots and interpreted using shape diagrams [19].

#### Comparison and interpretation of motion among individuals

In the second part of the study, we used the ‘modes’ identified with the k-means cluster analysis to explore and interpret inter-individual variability in feeding behaviour. This was done using summary plots with different colours for different ‘modes’:

- First, we calculated the individual frequencies of the ‘modes’ and summarised their variation using bar-plots.
- Then, using scores from the MAS PCA calculated in part I, we plotted PC1 against time (i.e., frame number) in each individual, and did the same with PC2, to compare changes in ‘mode’ in relation to time during feeding.
- We also repeated the PCA within each individual separately. To avoid confusing this series of 31 analyses with the one of MAS (part I), we employ the abbreviation iPCA and iPCs for the individual PCA and PCs respectively. Thus, with iPC1-2 scatterplots, we qualitatively compared motion patterns between the 31 individuals, as well as with the main pattern found in MAS (part I).
- Finally, we summarised and investigated individual variability in centroid position, cumulative foot angle, and CS in relation to feeding ‘mode’. For centroid position, we simply used scatterplots of its raw X and Y coordinates in each individual. For foot angle and CS, we summarised inter-individual differences using box and jitter plots, and also plotted each variable against time in every individual.

#### Correlational analysis of main descriptors of motion

To further explore variation, we investigated the relationships between the main descriptors of motion during feeding in *A. ricciae* (i.e., cumulative foot angle, centroid position, CS, total shape, and shape summarised in the main analysis of part I by PC1 and PC2) by calculating their variances in each individual. We used variances because we want to assess whether a large variation in one component of motion (e.g., changes in body size) co-occurs with large variation of another component (e.g., changes in body shape), indicating a connection. Variances were log-transformed using the natural logarithm to improve normality and used to compute pairwise Pearson correlations between variances of all six descriptors of motion. For multivariate shape, the sum of the variances of the shape coordinates was used as an estimate of total variance. For calculating and plotting the correlations, we employed the function *chart.Correlation* of the R package PerformanceAnalytics [32].

## Results

### Tangent space approximation

The correlation between MAS Euclidean shape distances in the tangent space and Procrustes shape distances is 1.000 and the regression slope 0.985. The largest pairwise Procrustes distance between any two of the 1674 frames is 1.16, which corresponds to 74% of the largest possible shape difference in the Procrustes shape space. However, 90% of frames of the main analysis have a pairwise Procrustes shape distance ≤ 0.40, which is 25% of the maximum possible distance in the Procrustes shape space. Thus, despite a poor approximation for the most distinctive shapes in the sample, all other statistics for assessing the goodness of the tangent space approximation suggest a good fit for the vast majority of the individual frames and, therefore, mostly negligible distortions for MAS after the tangent space projection.

### Range of postures during feeding and discrimination of ‘modes’

**Fig. 3:**
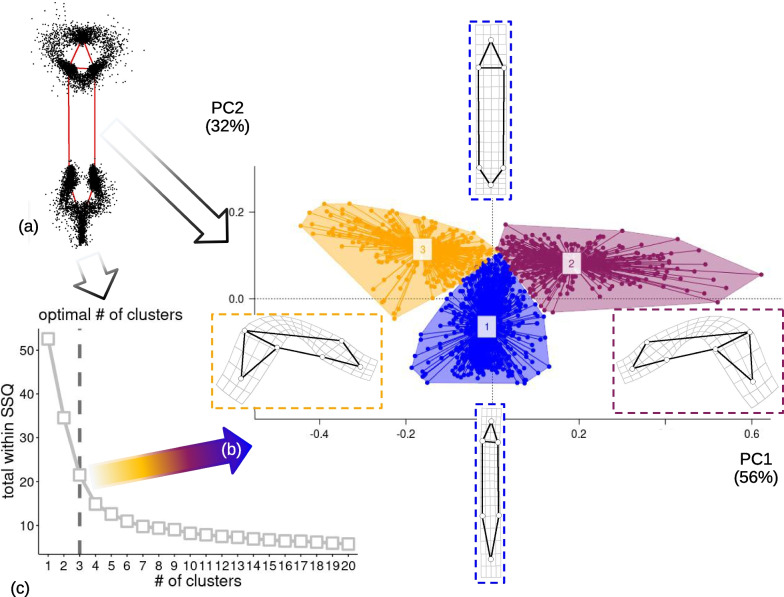
Main analysis: (**a**) Procrustes superimposed individuals in the MAS, with the mean shape emphasised using a red wireframe (the head of the animal is the triangle, in the upper part of the plot, and the foot is at the opposite extreme). (**b**) Scatterplot of PC1-PC2 (percentage of variance accounted for by a PC is in parentheses in this and other PCAs); the wireframe diagrams with deformation grids show the shapes at the opposite extremes of each PC; the main clusters ('modes' of postures) found using a k-means cluster analysis are emphasised using convex hulls and different colours (in this and all other figures: blue, for foot telescoping; orange or brick red, for bending to one or the opposite side). (**c**) Mean clusters SSQ *vs* number of clusters in the k-means cluster analysis

The first two MAS PCs of Procrustes shape (Fig. 3a) summarise almost all (88%) variation of posture shapes in the total sample. In the PC1-2 space (Fig. 3b), the observations form a Y shaped pattern, with the wide and elongated arms of the Y having mostly positive scores on PC2. The observations with negative scores on PC2, in contrast, tend to be close to zero on PC1, thus giving origin to the short stem that separates the long arms of the Y. The visualization of shape changes along PCs (shape diagrams in Fig. 3b) shows that PC1 corresponds to the animals bending to one or the opposite side, during feeding, whereas PC2 mostly captures the telescoping of the foot.

Searching for a parsimonious number of clusters (Fig. 3c) suggests that the major drop in average variation (SSQ) happens with three clusters. Thus, we did the k-means cluster analysis of the MAS shape data with three clusters and, regardless of the initial number of random steps, consistently found the same groups of 1,006, 350 and 318 frames (i.e., respectively 60%, 21% and 19% of the total frame number). In all figures, these groups are consistently coloured in, respectively, blue, brick red, and orange. By plotting the groups on PC1-2 (Fig. 3b), it is evident that the main cluster (blue symbols) describes a mode of motion where the body is straight or moderately bent and the telescopic foot varies in length (foot telescoping). The other two groups (orange or brick red), which split the remaining observations almost precisely in two halves, are the main parts of the arms of the Y, which, as anticipated, correspond to animals bending to one or the opposite side, while the foot is retracted (i.e., short).

### Variability among individuals

**Fig. 4:**
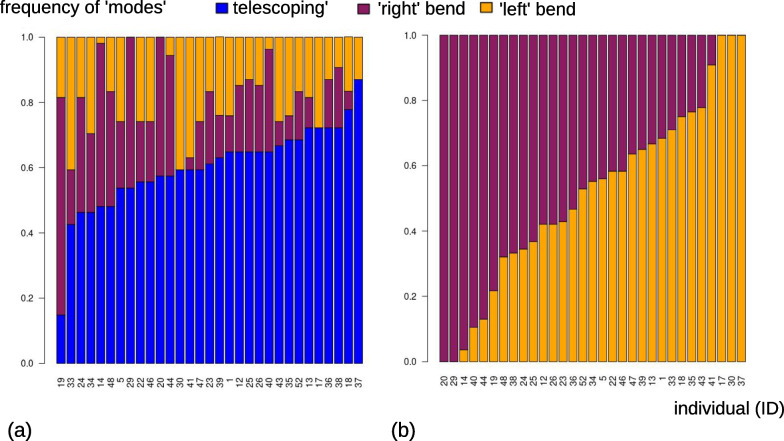
Frequency of the three ‘modes’ of motion during feeding: (**a**) includes all three ‘modes’ and has individuals ordered according to increasing frequency of foot telescoping; (**b**) focuses on bending with individuals ordered according to increasing frequencies of left bending

Bar plots of the frequencies of the three ‘modes’ (Fig. 4) indicate large differences between individuals. Some (left side of Fig. 4a) perform less foot telescoping (short blue bars), although in most individuals telescoping accounts for at least 40% of motions, and others do mostly foot telescoping with some occasional bending to one or both sides (orange or brick red bars). If we focus on bending (Fig. 4b), we also find large variability: there are animals who only, or almost only, turn to one or the opposite direction (left and right extremes of the bar plot in Fig. 4b) and others whose bending is almost perfectly split 50:50 between one or the other direction.

**Fig. 5:**
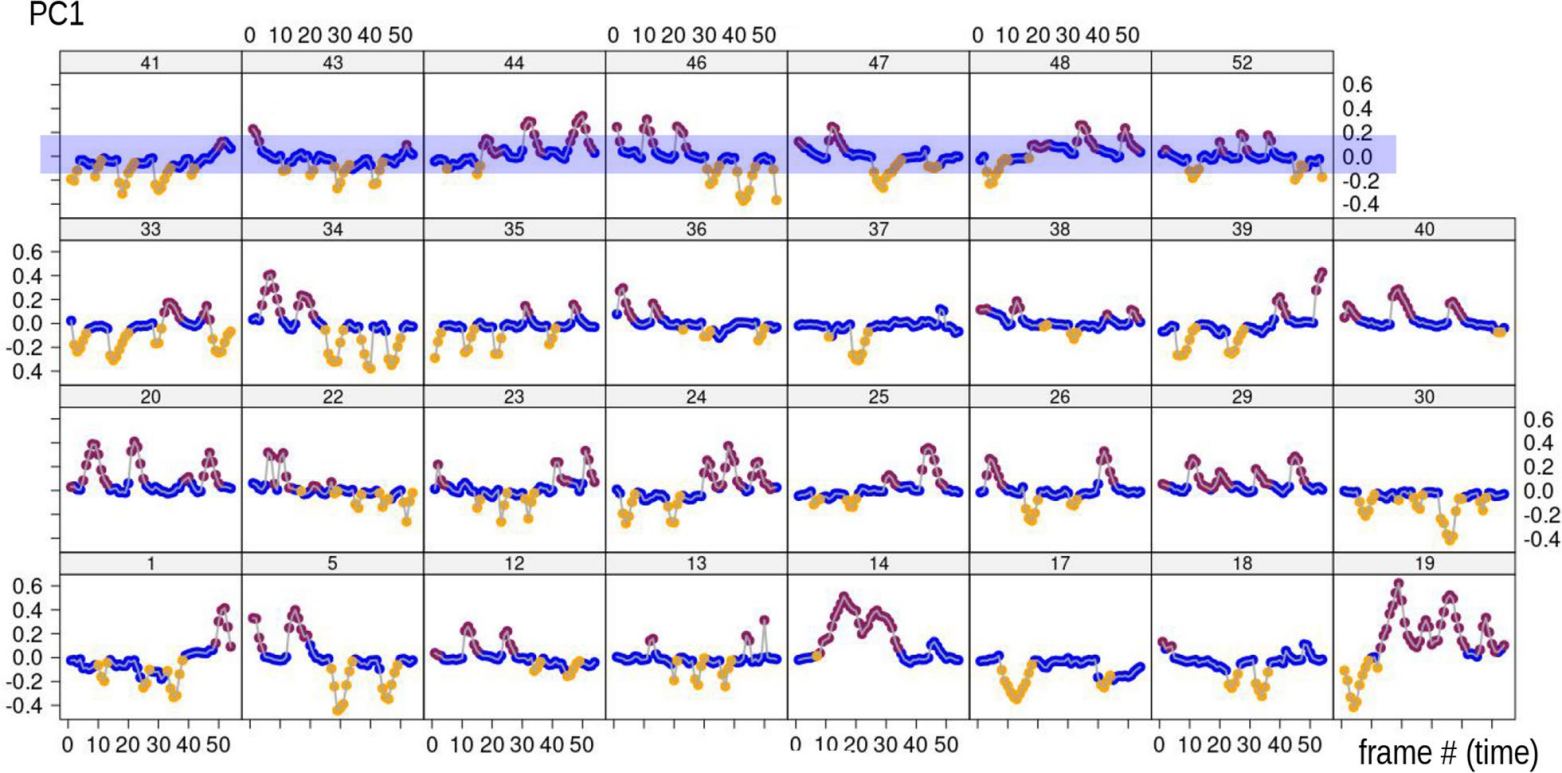
PC1 of MAS shape (vertical axis, 56% of total variance) plotted against time (frame number, horizontal axis) within each individual (individual ID—identifying number—shown above each plot in this and the next figures). The blue transparent box in the top series of scatterplots emphasises, as an example, the near constancy (≈ zero) of PC1 scores for foot telescoping

**Fig. 6:**
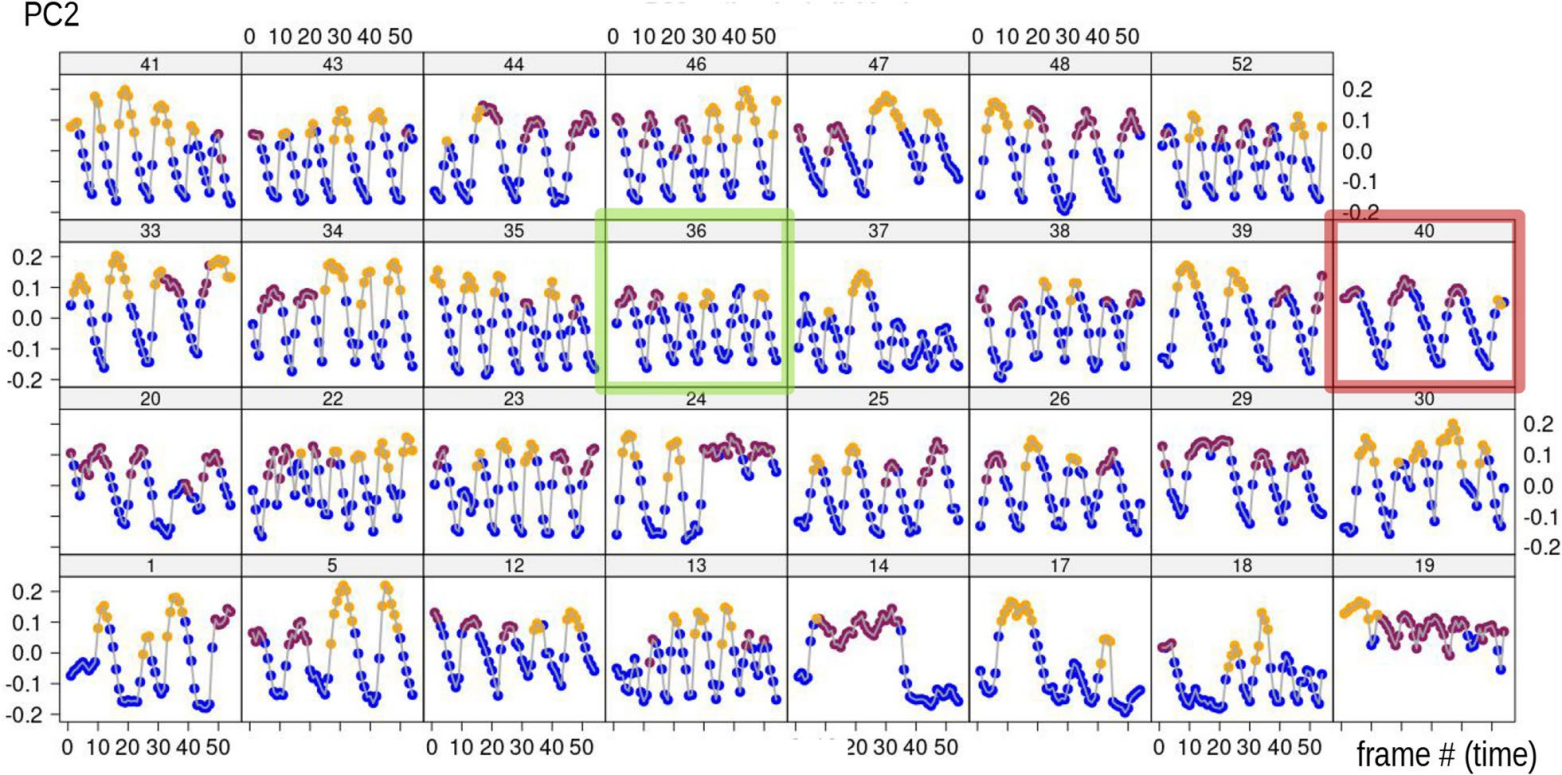
PC2 of MAS shape (vertical axis, 32% of total variance) plotted against time (frame number, horizontal axis) within each individual. Coloured frames in this and other figures emphasise individuals whose description is used as an example in the main text

**Fig. 7:**
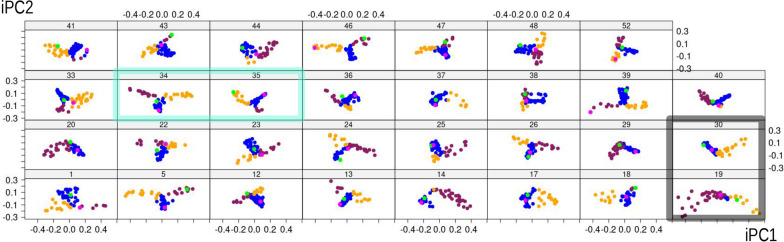
Individual principal component (iPCA) scatterplots of iPC1 vs iPC2. Variance accounted for by iPC1 ranges between 51 and 81%, and for iPC2 between 13 and 43%, with the cumulative variance of PC1-2 being 87–95%. In this figure and the next one (Fig. 8), the first and last (i.e., 54th) frames of each individual are emphasised using, respectively, the colours green and magenta

Figures 5, 6 plot, respectively, PC1 and PC2 scores from the MAS PCA against time in each of the 31 individuals in order to explore variability in modes in the sample. We stress that these are the scores of the PCA computed using the 1,674 frames dataset. Later, inter-individual variability is also assessed by repeating the PCA one individual at a time (iPCAs in Fig. 7). Thus, in Figs. 5, 6, it can be observed that in all individuals during foot telescoping (blue symbols) PC1 is close to zero, and, thus, scores form approximately horizontal blue lines (Fig. 5). When an individual bends, the trend line goes up or down, which corresponds to the orange (negative scores) or brick red (positive scores) peaks in the plot (Fig. 5). Figure 6, showing PC2 *vs* time, is also interesting, as it suggests a periodicity and, therefore, a sequence of repetitions in the behaviour. This happens because PC2 captures foot telescoping, but also the very beginning of bending to one or the other side (larger positive scores in Fig. 3b). For instance, in individual 40 (dark red frame in Fig. 6), the first frames of feeding capture an animal that was bending to one side (brick red), but soon switched to foot telescoping, before bending again to the same side. This was followed by more telescoping and bending to that same side, to only later, in the last frames of the sequence, beginning to bend to the opposite direction (orange). If we compare individual 40 with 36 (green frame), there is a similar periodicity, but periods are shorter (closer peaks) and bending switches to the opposite side (orange) after just two bends (brick red) in the other direction. The closer, more numerous, peaks of 36 suggest a faster pace of foot telescoping, although that probably happens with less pronounced contractions of the foot (smaller amplitude in terms of PC2 scores) compared to 40.

Figure 7 focuses on each specimen separately and, therefore, summarises individual-specific patterns of feeding with 31 separate shape PCAs (iPCAs). In this figure, by plotting ‘modes’ found in MAS (part I of the methods), one can compare differences and similarities among individuals and in relation to the pattern found in the population sample (Fig. 3b). If the Y-shaped pattern found in MAS (Fig. 4) is also present in the analysis of each individual one at a time, this indicates that the entire behavioural sequences with all three modes of motion are replicated by each individual every time it feeds. Contrary to this expectation, however, it is clear that there is a large amount of variation depending on the specific individual. For instance, individuals 34 and 35 (emphasised with a cyan frame) are both characterised by a Y shaped pattern of body shape change during feeding, reflecting the full range of postures in the main analysis, although the Y is turned approximately upside down in 35. Also, 35 does most of the bending in one direction (orange), whereas 34 bends about as often to one as to the other side (see also the frequency bar plot in Fig. 4b, with 35 to the right, orange, extreme and 34 approximately in the middle). If this observation is coupled with that of Figs. 5, 6, one can appreciate another difference in feeding behaviour between these two individuals. 34 first bent twice to the same side (with some foot telescoping between the two bends) and only later switched to bending in the opposite direction, which it did three times. In contrast, 35, after initially bending multiple times to the same side, later alternated one and the other side, as suggested by the three orange peaks followed by one brick red, one orange, and another brick red peak. Some other individuals, at least in relation to the short time of the feeding sequence available for the analysis, show a different, non-Y shaped, pattern, which we exemplify using individuals 19 and 30 (Fig. 7, grey frame). Individual 19 did almost no foot telescoping (see also Fig. 4a, where 19 is the first to the left), mostly turned in one direction (brick red) and only rarely, at the very beginning of the recording (Figs. 5, 6), in the opposite one (orange). Individual 30, in contrast, did more foot telescoping (blue) but only bent to one and the same side (Fig. 4b, right extreme), with a fairly regular alternation of foot telescoping and bending (Figs. 5, 6).

**Fig. 8:**
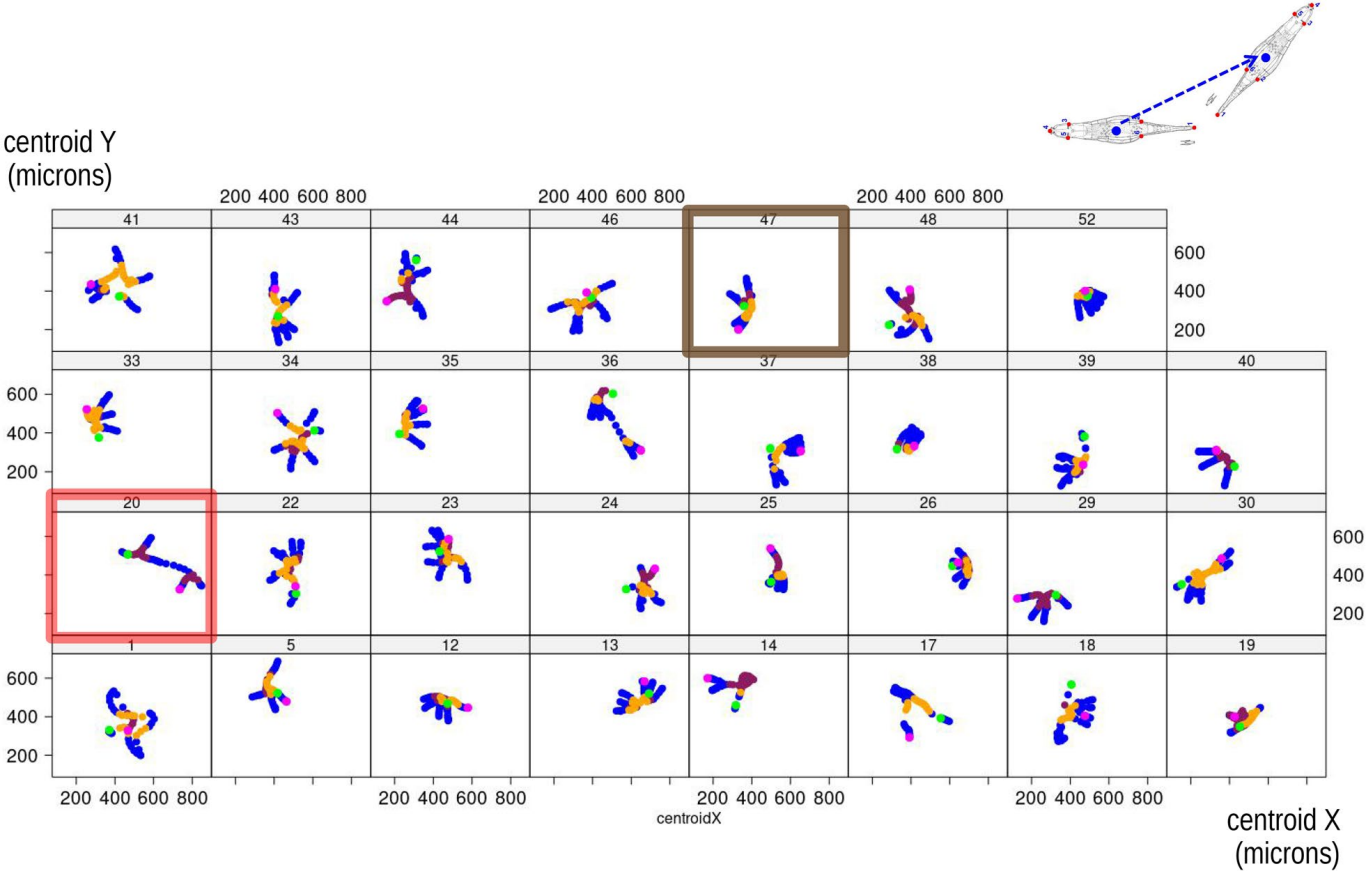
Changes in raw coordinates of the centroid position (illustrated in the vignette in the upper right corner) during feeding of each individual

Tracking the centroid (Fig. 8) of the landmark coordinates of an individual provides some further information, complimentary to shape data. It is possible to relate feeding ‘modes’ to shifts of the centroid due to changes in posture and organismal motion as a whole. For instance, individual 47 (brown frame in Fig. 8, but also shown in Fig. 2) approximately shows three or four straight lines (trajectories) originating from the same spot. Bending (mostly orange) is close to the common origin of the lines and foot telescoping (blue) happens at the opposite, divergent, tips of the trajectories. This suggests an individual that had the tip of the foot attached to a specific spot and fed by alternating foot telescoping and bending. In this individual, bending occurred more frequently to one side (orange—Figs. 5, 6), with the different directions of the centroid trajectories indicating two main body rotations followed by a smaller one (see also Fig. 2a). The centroid plot of individual 20 (red frame in Fig. 8), in contrast, indicates a somewhat different pattern, in which the animal first fed in one spot (again with a cycle of telescoping and bending to one side) and later moved to a slightly different one, to the left, where it restarted the cycle. As the straight line between the two spots is blue, that means that individual 20 moved to the new spot by foot telescoping.

**Fig. 9:**
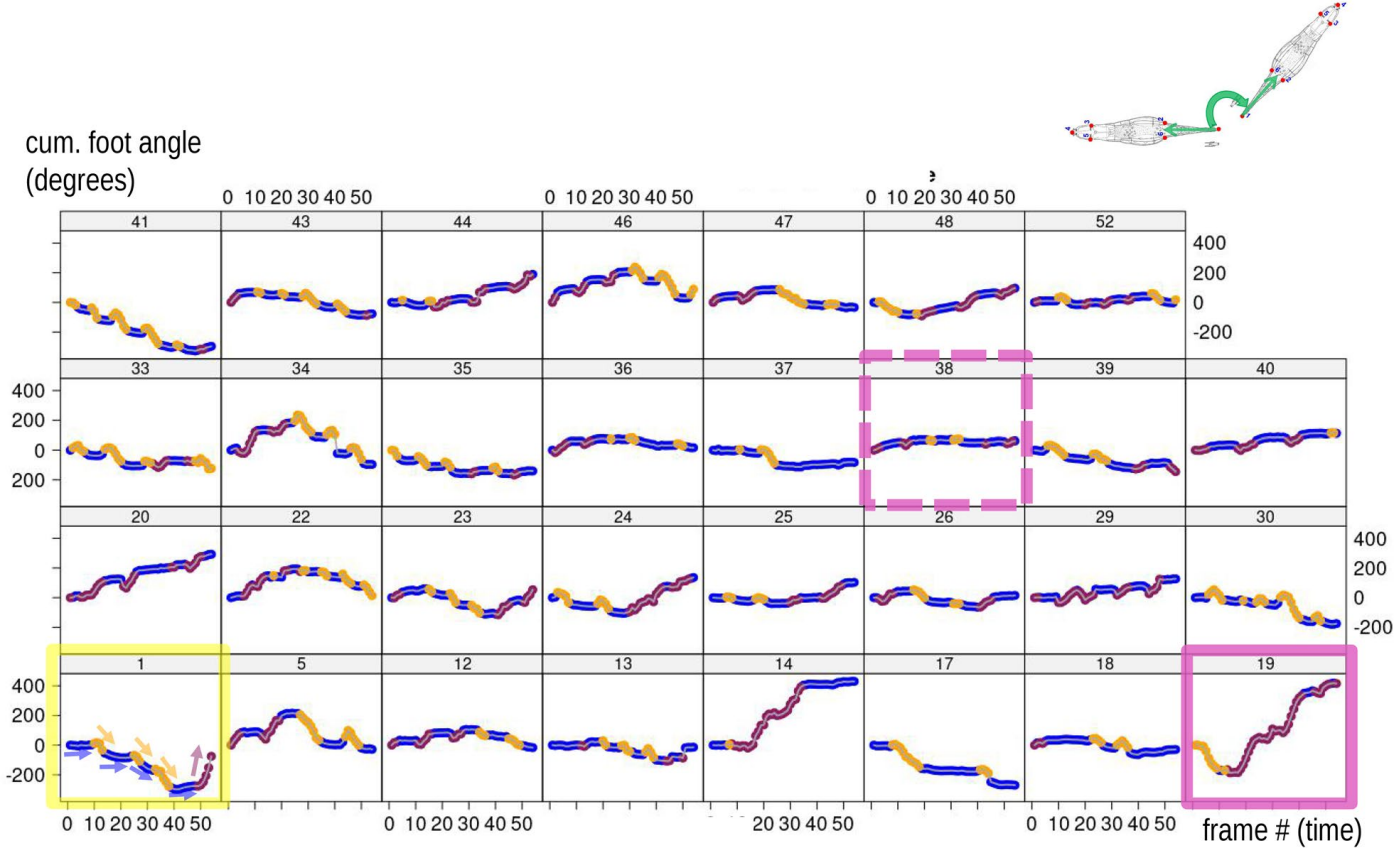
Cumulative foot angle (exemplified in the vignette in the upper right corner) plotted against time in each individual

**Fig. 10:**
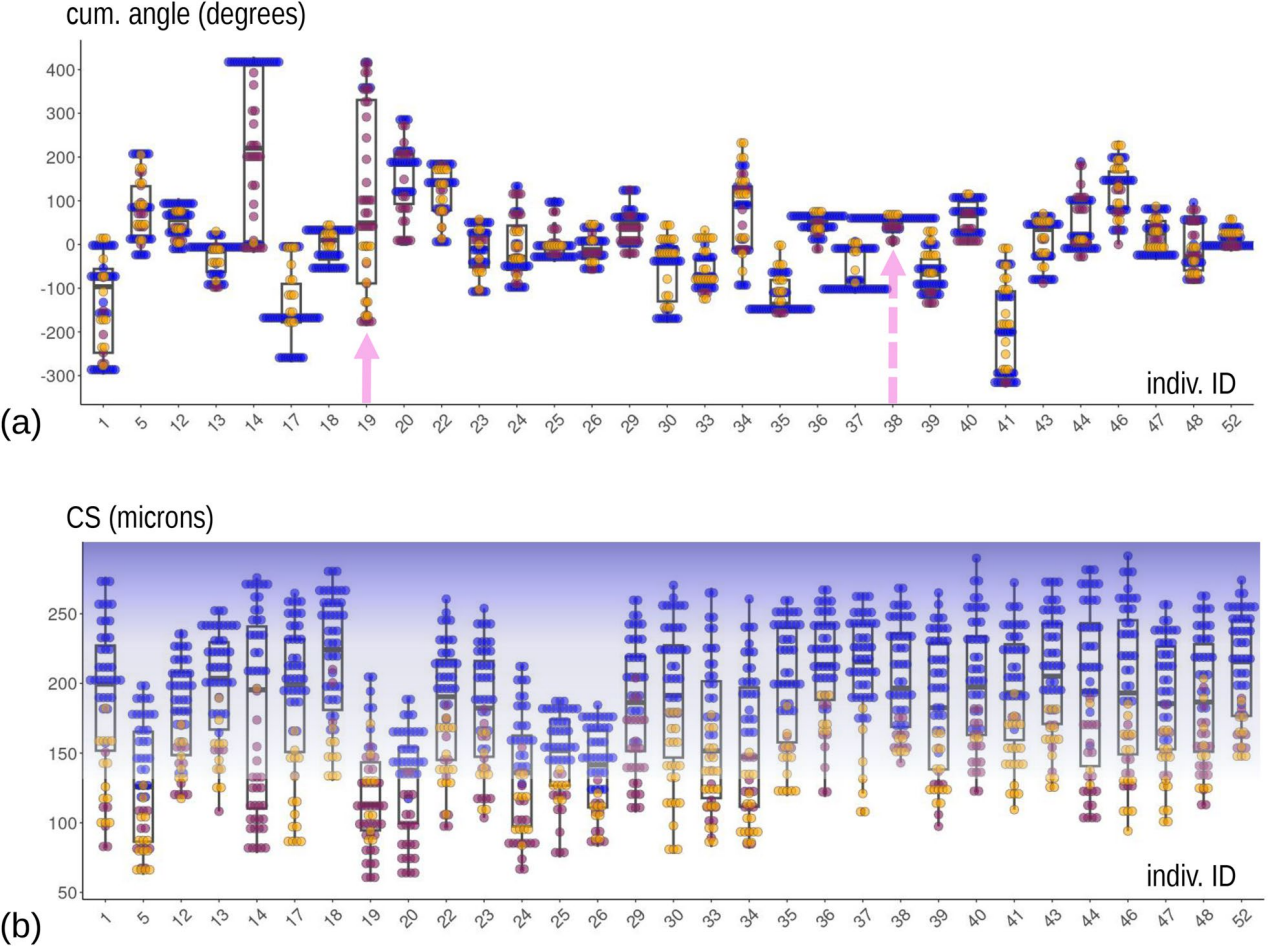
Box and jitter plots of cumulative foot angle (**a**) and CS (**b**). See main text for explanations of arrows (**a**) and background colour (**b**)

The cumulative foot angle also varies widely among individuals (Figs. 9 and 10a). This is easy to appreciate using two examples from these figures, that are at the opposite extremes of the range of variation: individuals 19 (solid pink frame/arrow) and 38 (dashed pink frame/arrow). Individual 19 shows large rotations of the foot, turning first more than 180° anticlockwise and then reversing the direction to end the sequence with an overall clockwise rotation of almost 400 degrees clockwise compared to the first frame. The wide rotations of the foot are concomitant with a prevalence of bending of the body (orange and mostly brick red), with rare foot telescoping (blue). In contrast, individual 38 only modestly rotates its foot and mostly does foot telescoping (blue), rarely bending (orange/brick red). Both Fig. 9 and 10a suggest that foot telescoping happens with no or modest rotations of the foot. Indeed, the blue ‘lines’, made by consecutive frames, are almost horizontal in Fig. 9, but tend to go up or down during bending (orange/brick red), as emphasised for individual 1 (yellow frame) using arrows. Similarly, in Fig. 10a, there tends to be overlap in angles, and thus no rotation, during foot telescoping (wide horizontal scatter of blue circles), whereas bending (orange/brick red circles) generally show a concomitant large variation (vertical scatter) in cumulative foot angles.

**Fig. 11:**
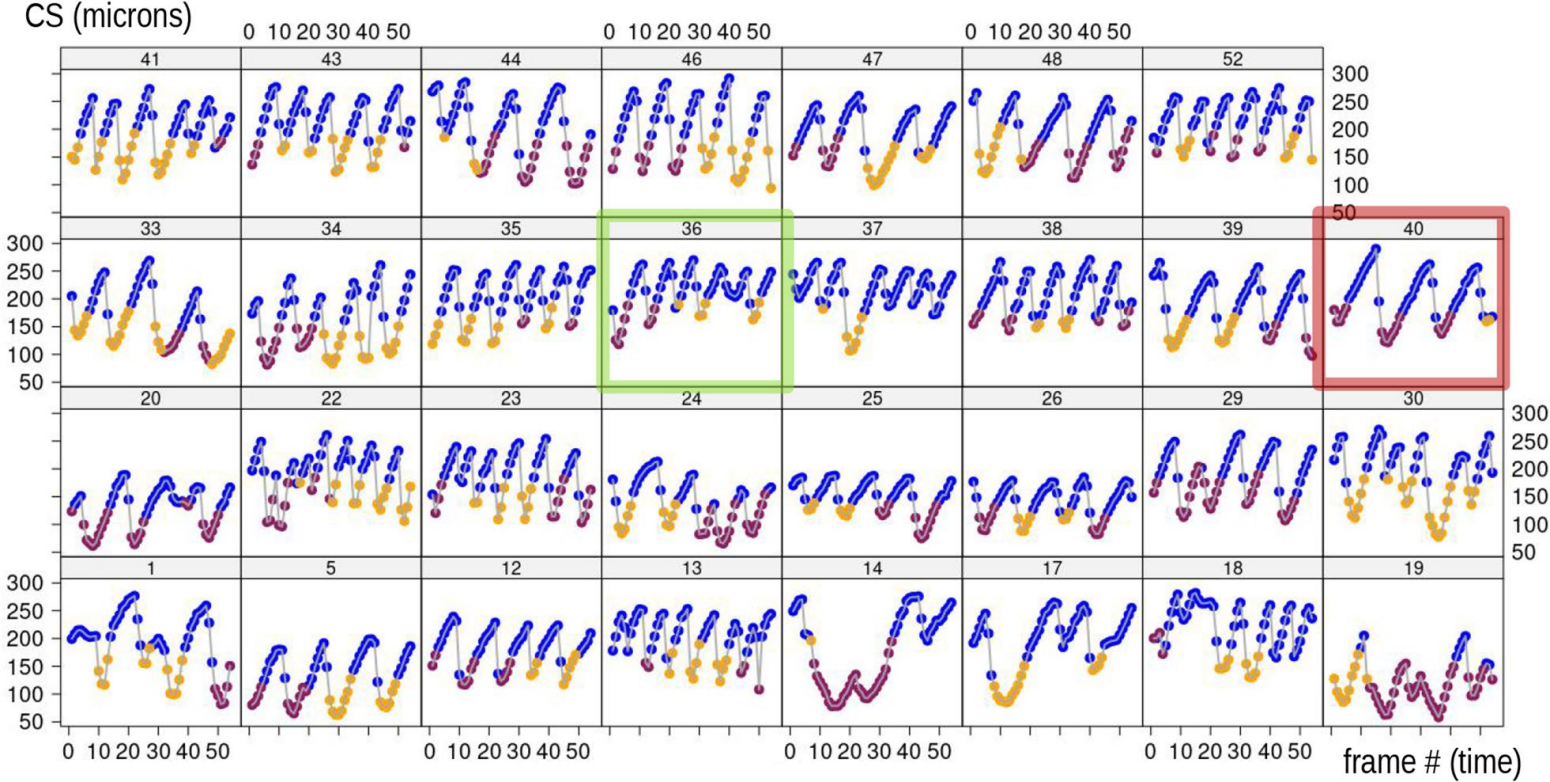
CS plotted against time in each individual

Finally, Figs. 10b and 11 show what happens to body size (CS of the configuration) during feeding. Size varies hugely within individuals and more moderately between them. Larger CS is characteristic of foot telescoping, as evidenced by the dominant blue colour in the upper part of the plots. Bending also implies large variations in body size, but these happen at smaller values of CS (lower part of the plots). As with PC2 *vs* time, CS, when plotted against time (Fig. 11), also shows periodicity and, this, suggests a repetition of cycles of stereotyped motions with different ‘speeds’ depending on the individual (e.g., compare again individuals 36 and 40, as in Fig. 6, emphasised using respectively green and dark red frames).

### Correlational analyses of motion descriptors

**Fig. 12:**
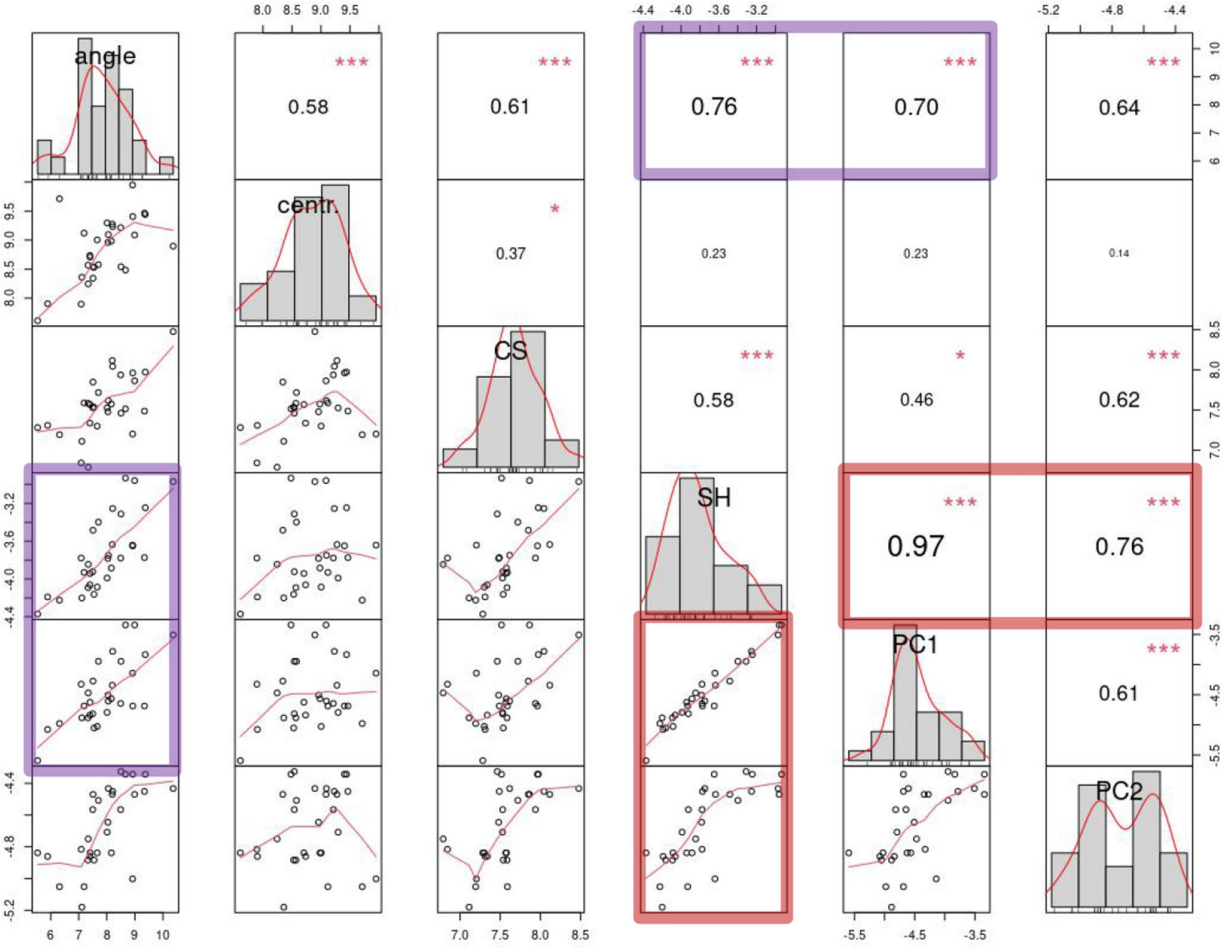
Relationships between log-transformed individual variances (i.e., the variance of a variable – e.g., foot angle–across the 54 frames of each individual) in the main descriptors of motion after removing individual 19, a strong outlier especially for its very low PC2 variance (as shown in Fig. 9, this is a consequence of 19 doing almost no foot telescoping, which is the ‘mode’ captured by PC2; thus, when 19 is included, it causes a likely spurious drop in the correlation of variance in PC2 with variance in total shape). Upper diagonal: pairwise Pearson's correlations, with significant values shown using larger fonts (the larger the font, the lower the p-value). Lower diagonal: pairwise bivariate scatterplots with a fitted trend line in red. Diagonal: frequency histogram and abbreviated name of the variable (angle = cumulative foot angle in degrees; centr. = centroid of raw coordinates (microns) used to track an individual position; CS = centroid size (microns); SH = total shape; PC1 or PC2 = shape captured by respectively PC1 or PC2 in MAS)

In the correlational analyses of individual variances in motion descriptors we focus on the largest correlations, which are supported by scatterplots suggesting clear collinearity and modest scatter. Figure 12 shows that large variations in shape (both total and in PC1 of the main analysis) occur together (r ≥ 0.7) with large variations in cumulative foot angle. Also, variation among individuals in PC1 is an excellent proxy for total shape inter-individual variation (r = 0.97). That most shape variance is accounted for by PC1 is a truism in a PCA. However, in our analyses, variance in PC2 has a large correlation (r = 0.76) with total shape variance, which is reasonable since PC2 has the second highest variance in the PCA. In contrast, it is more interesting that body bending, captured by PC1, co-occurs with large rotations of the foot (r = 0.70), a finding consistent with the observations made in Fig. 9.

## Discussion

Movement is one of the defining features of animals and a central aspect in the study of animal behaviour. Our research exemplifies the application of Procrustes motion analysis in a microscopic freshwater invertebrate species. With just six anatomical landmarks, we could describe motion during feeding in the bdelloid rotifer *Adineta ricciae* in a simple two-dimensional space, find the main ‘modes’ of motion and combine information on shape changes with data on position, rotation, and body size to better capture the complexity of movement during feeding in this species. With these analyses, we confirmed that foot telescoping is used not only for moving during the displacement phase between nearby feeding spots,^7^ which may also occur by gliding using ciliary beating, but also while an individual feeds in a specific place. Telescoping generally happens with the body kept relatively straight, whereas bending (to one or the other side) is mostly done with the foot retracted, while the head and trunk turn, so that the animal body looks ‘squeezed’ and smaller. During bending, with the foot usually firmly attached to the substrate, an individual often rotates the foot, producing angular changes that can be small or very large, as well as clockwise, anticlockwise or a mix of the two. Exploring variability in our sample also showed that the pattern of motion is generally similar, but the frequency of ‘modes’ varies widely across individuals and, at least in a short sequence of frames, the full repertoire of motion may not occur. The application we presented is largely graphical and exploratory but has the advantage of simplicity and can be easily extended to broader and more complex types of research on animal motion in small invertebrates.

### Assumptions, methodological issues and open questions

Before focusing the Discussion on the methods we used, its main results in *A. ricciae*, and the potential future directions, we clarify some methodological issues in our analysis, starting with an important assumption. As we do not know the distribution of food in the experimental settings and it is difficult to accurately observe the behaviour of highly active but tiny organisms such as rotifers, we cannot be completely sure of what they are precisely doing when they stop and begin rotating, bending and telescoping. However, from what is known of the biology of this group, in which animals feed by scraping off microorganisms from the surface of the substrate [7], assuming that they are searching for food when they keep the foot attached to a substrate seems a reasonable and parsimonious explanation. Indeed, even if the slide was cleaned before placing the animal in a clean drop of water for the observation and videorecording at the microscope, microorganisms or tiny scraps of food (organic debris etc.) might have been present.

As anticipated, the goodness of fit of the tangent space approximation is an important factor to consider in Procrustes motion analysis, because, unlike static data in biology (e.g., mammal crania in evolutionary comparisons [23]), movement tends to produce a very large variations in shape. This is the case in *A. ricciae* during feeding movement, as the largest shape distances between frames of MAS span almost ¾ of the largest possible difference one might find given this specific set of landmarks. However, in our dataset, such large distances only occur in a relative minority of observations and, thus, their distortion is unlikely to influence the robustness of the main findings. Nonetheless, if one is very concerned about this issue or has data with a bad tangent space approximation for most observations, there are statistical methods that can be applied based on Procrustes shape distances, as shown by Adams & Cerney [10].

For *A. ricciae*, a more important problem might be the orientation of an individual during feeding in experimental settings. Because the individuals move in the film of water between a slide and a coverslip, this may constrain or bias the behaviour. However, as in natural conditions they move in the narrow water-filled spaces between particles of soil sediments or on the subtle film of water covering the leaves of mosses, our laboratory settings may represent a relatively good approximation. No natural surface could be as flat as the slide, but the flatness of the slide removes potential distortions in the observation of the movement behaviour. Thus, probably, the main difference between the experimental conditions is that the animals move across a flat horizontal space instead of a more complex three-dimensional environment as in the wild.

Another potential issue in the videos is that the resolution of the images may not be high enough to distinguish the dorsal from the ventral side. In our sample, the resolution was adequate for us to exclude individuals that were recorded in ventral rather than dorsal view. However, without this type of information, bilateral landmarks (2, 3, 5, 6) might have been swapped in some individuals, so that it would become impossible to say whether an individual is bending to the right rather than to the left. Not being able to identify if the view is ventral or dorsal would, thus, be a clear limitation in a behavioural study. Nonetheless, in these small bilaterians, it is reasonable to expect that left and right sides are functionally equivalent during motion and feeding. Indeed, although in a limited sample we cannot say with total certainty whether there is a preferential direction in which animals turn or bend, it looks likely that there is not since our sample of motions in *A. ricciae* displays neither right- nor left-handedness during search for food. This is suggested by the similar overall frequency of bending to the right or left in our dataset of individuals filmed from their dorsal view. Furthermore, an additional clue for the effect of right *versus* left direction comes from the summary plots of motion of each individual. Despite the short series of frames, a good proportion of individuals in the main sample shows a distribution of postures (Y-shaped and roughly symmetric) on the first two iPCs (Fig. 7) similar to that of the total sample. This confirms that bending to one side happens about as often as bending to the opposite one.

In spite of broad similarities, our results show that there is clear individual variability both in patterns and lengths of the trajectories (Figs. 4, 5, 6, 7, 8, 9, 10, 11). This is in contrast to the highly reproducible patterns found in the nematode *Caenorhabditis elegans* (Maupas, 1900) [4] and to some extent in fruit files [33]. Variability may look counter-intuitive in bdelloid rotifers, which reproduce asexually. However, phenotypic variability in bdelloid rotifers is known also when comparing mothers to their clonal daughters [34, 35]; even if asexual, offspring production in *Adineta* is a process that is able to introduce genetic variability [36] on top of differentially expressed epigenetic inheritance [37] of different daughters of the same mother. Thus, differences in how an animal feeds could be genetic, epigenetic, related to subtle environmental variability, or simply due to different age or timing of the animals. For instance, the distribution and amount of microorganisms at a feeding spot almost certainly varied from individual to individual, given that different glass slides used for observation, even if clean, may still contain impurities. If we had longer sequences, we would expect that minor differences would disappear. An asymmetric pattern, where an individual bends only to one side, is likely to be due to sampling error in short series of frames, as the same specimen might have bent to both sides, if it had fed for a longer time in the same spot. The duration of feeding in one or other spot should depend on the abundance and distribution of food, but also latent, and less obvious, factors, maybe also intrinsic in each animal, given that food was scant or almost absent in the slides we used for the observations. Unfortunately, as the animals keep moving quickly and usually spend no more than a fraction of second in the same feeding spot, it is difficult to obtain longer series of frames. Likewise, as already stressed, it is hard to track an individual as it moves away from a feeding spot in order to record multiple feeding sessions.

Although it did not involve the use of superimposition methods, using a K means cluster analysis to identify motion types is well exemplified by Park et al. [30], who employed it to distinguish different techniques for load transfer and lifting in humans. As in our study, to infer an appropriate number of clusters, they employed a combination of ordination methods (multidimensional scaling, in their case) and K means cluster analysis. A disadvantage of this approach, however, is the presupposition of the existence of different types of motions [30], as well as the risk of creating artificial discontinuities in what is, in fact, a continuous range of variation. Thus, ‘modes’ too have a degree of arbitrariness. This is especially obvious at the boundary between clusters, where observations in one cluster are similar to the neighbouring ones in a different cluster, so that whether they end up in one or the other might have more to do with chance than with substantial biological differences. In order to appreciate this type of uncertainty, a researcher could bootstrap the data and repeat the analysis many times to estimate confidence intervals around the observed frequencies of ‘modes’. In our study, for instance, bootstrapping individuals 1000 times and re-rerunning the main analyses confirms that results are robust. The average standard error is 8% relative to the number of frames classified as telescoping or bending to the left or right in MAS (i.e., as reported in the Results, respectively 1006, 318 and 350 frames) and the average confidence interval (estimated using the 10th and 90th percentiles of ‘mode’ counts in the bootstraps) is about ± 10% of the observed counts for each ‘mode’.

The number of groups in the k-means analysis is also based on an arbitrary decision. This is true even when supported by numerical analyses aimed at making this choice less arbitrary, as in our study. The selection of the method to decide a parsimonious number of groups is, itself, subjective. Besides, in our specific case, the sharpest drop in average within cluster variation happens with three clusters, but there is a further, small but visible, drop with four clusters too. We decided to use three instead of four to make the description simpler. Had we employed four clusters, a first difference would have been that the initial stage of bending, when it is minimal and the foot is very short, would have ended up in the fourth cluster instead of being (usually) part of one of the two capturing bending. A second, more important difference would have been that variation on PC2 (i.e., foot telescoping) would have been split in two, with one group made of observations with a more extended foot and the other consisting of those with a shortened foot. Yet, foot telescoping is a single type of behaviour, and it does not seem useful to divide it into subtypes. Nonetheless, if a researcher privileges detail over simplicity, he/she might want to experiment with more rather than fewer clusters.

Specific aspects of the mathematical description of motion are also a consequence of the choice of morphometric descriptors (the landmark configuration, in our work) and analytical method, and should be interpreted with caution. For instance, as pointed out by Stephens et al. (p.5 [4]), “because modes [in their study] are eigenvectors of a covariance matrix, their instantaneous amplitudes are not linearly correlated, but this does not mean that the dynamics of the different motions are completely uncoupled”. In our analysis, this statement can be translated into the observation that bending to one or the other side also involves a small amount foot telescoping and some of the frames mainly capturing foot telescoping might show a degree of head turning to the left or to the right. Indeed, our approach is complementary to the method of Stephens et al. [4], as in both studies the aim was to simplify the description of behaviour in a way that is largely data-driven. They used an eigenshape analysis to quantify changes in the central curve describing the body (which is analogous to employing a PCA of the measurements of the curvature of the body axis). Yet, as in our analysis using landmark-based Procrustean morphometrics, they also discovered similar stereotyping of motion with ‘attractors’ corresponding to the main axes of variation in postures. Likewise, we could describe feeding in *A. ricciae* as a behaviour with two ‘attractors’, which are the main directions of shape change. One ‘attractor’ describes foot telescoping and the other, slightly less frequent but with larger variation in shape, is made up of two ‘modes’, which are the bending of the trunk to the left or to the right. Thus, it is true that the landmark configuration we adopted is an arbitrary but parsimonious compromise to achieve accuracy using a limited number of morphometric descriptors. However, the number of PCs capturing most variation in motion during feeding is a data-driven outcome of the shape analysis. That two PCs are enough to describe the main pattern of changes in posture in *A. ricciae*, whereas the “eigen-worm” analysis of Stephens and colleagues required the first four PCs, might be due partly to methodological differences but also to real differences in the complexity of the behaviour being measured. When the number of morphometric descriptors is increased, as happens with techniques that are designed to measure entire curves, more details are captured and those might better quantify a complex behaviour. However, landmarks, unlike outlines [38, 39], have the advantage of a well-defined and precise anatomical correspondence and, thus, seem appropriate when a small configuration can provide an effective quantitative description of a relatively simple movement, as happens with feeding in *A. ricciae.*

### Further analyses and alternative complementary approaches

Procrustes motion analysis is one among several approaches to measure motion in behavioural studies [1, 2, 5, 40] and has different options in terms in how it is applied. The method, as originally proposed, is well described by Piras and colleagues (p. 5): “This approach is basically the multiple alignment of shapes ordered in a temporal sequence … a ‘‘trajectory’’ has itself a shape, a direction, and a size, quantified as the sum of phenotypic distances among an ordered sequence of shapes along a trajectory” [41]. Thus, in the original framework proposed by Slice [12], shapes in a series of frames capturing motion become themselves a trajectory in shape space whose size, shape, and orientation can be analysed and compared. One clear advantage of this approach is that each individual is represented by a single trajectory instead of multiple non-independent shapes.

In our focal species *A. ricciae*, however, trajectories are not always easy to compare. This is not only because it is hard to have a comparable starting point in the recording of motion, but also because, as shown in Fig. 7, there is a wide range of variability in the shape of the trajectory. If, at a population level and in several individuals, the trajectory is Y-shaped, in several others has a different shape. For instance, if an animal is bending only to one side (as it happens in individual 30) or does little foot telescoping (as in individual 19), one arm of the Y may be missing or the stem be almost absent. These differences could be partly due to the short recording time, which does not allow capture of the full range of postures during feeding but, at least at a specific spot, could also be real variation in behaviour. Because *A. ricciae* is hard to follow as it moves on a microscope slide, longer series of frames might be obtained by relaxing the requirement of a single session of feeding, of the same duration, in a precise spot and by appending different, non-consecutive feeding sessions of the same individual.

The analysis of motion trajectories could take advantage of the results of the search of ‘modes’. Within a given ‘mode’, trajectories will be simpler and potentially more comparable, but also shorter, which could require exclusion of some individuals. ‘Modes’ could also be summarised by averaging shapes within individuals and ‘mode’, so that each individual is represented, in our example using the bdelloid rotifer *A. ricciae*, by three sets of variables (if all three ‘modes’ were present when it fed). The resulting summary could be used to compare, for instance, foot telescoping (or another ‘mode’) in one sample with the same ‘mode’ in a different sample (clone, population, species, treatment etc.). This is a rather unsophisticated and inelegant approach to circumvent the non-independence of the observations in a sample, an issue that must be born in mind to avoid naive tests of group differences that mix variation among individuals with variation among individual frames. Thus, averaging ‘modes’ might help mitigate the problem of non-independence, but has the disadvantage of missing within individual variation and of oversimplifying the description.

Procrustes motion analysis also provides other types of useful quantitative information relevant to a wide range of potential studies. We showed that, after having detected the main ‘modes’ of motion in a specific behaviour, frequencies of ‘modes’ can be computed for the individuals in a sample. These could be compared with similar data from other populations or experimental treatments. Shape, size, and other descriptors can be also analysed in relation to time, as with time series. Indeed, the simple graphical comparison of, for instance, PC1 or PC2 *vs* time has allowed us to describe in detail some of the main individual differences. Complementing this information with the position of the centroid, as well as the cumulative foot angle and CS, potentially adds further insights into the feeding behaviour of *A. ricciae*. Thus, as mentioned, we were able to show that foot telescoping is used both when standing in a specific spot, as well as when moving away from one spot. Body bending and changes in foot angle, in contrast, mostly happen when the animal is in one spot and is, likely, trying to find and collect most of the food available nearby.

**Fig. 13:**
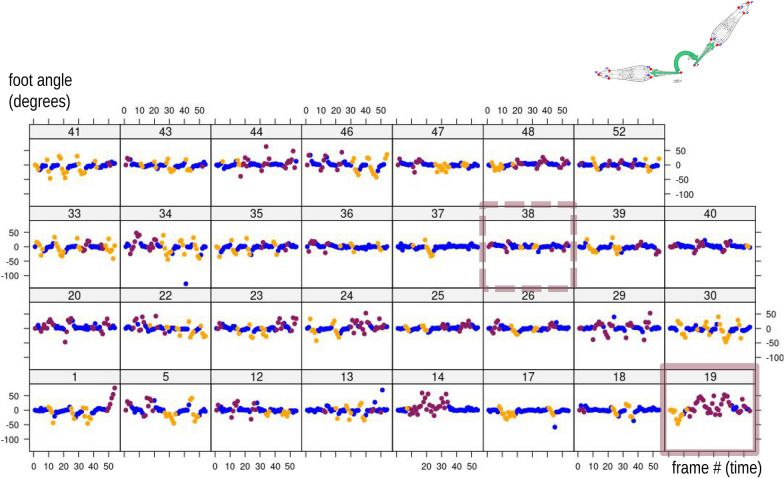
‘Instantaneous’ changes in foot angle (i.e., computed from one frame to the next, unlike in Fig. 9, which shows cumulative foot angles) plotted against time in each individual

Cumulative foot angles measure how much the animal rotates during feeding relative to the initial orientation. Because Fig. 9 clearly suggests that foot rotation mostly occurs as an individual bends to one or other side, whereas there is little rotation during telescoping, a reasonable guess is that rotational velocity (i.e., the angle that the foot covers over time) is higher during bending and almost constant during telescoping. That this is indeed the case can be convincingly seen if, for each individual, we plot the ‘instantaneous’ angular change in foot orientation from one frame to the next (Fig. 13). Because frames are equally time-spaced, this is like plotting angular velocities. With few exceptions, the angle changes minimally during foot telescoping (almost horizontal series of blue circles), whereas it varies a lot as an animal bends (orange/brick red circles usually above or below the horizontal line formed by the blue symbols). Here too, inter-individual variation is evident with, at opposite extremes of the range, specimen 38, that does almost no bending and, therefore, hardly shows any variation in angles (almost zero angular velocity), and specimen 19, which bends to the right or left almost all the time and displays high angular velocities (i.e., large deviations from zero, the original orientation of the foot). As expected, because angular velocity and cumulative angles are different ways of conveying similar information on orientation, their individual variances are highly correlated (r = 0.71) and both covary mostly with total shape and PC1 variances, which mainly relate to the amount of bending.

Procrustes motion analysis is a flexible approach and can be modified for specific purposes, as in [41]’s quantification of heart shape and volumetric changes in healthy and pathological individuals using ‘linear shifts’ or in the recent work of Laird and colleagues [42], who measured human jaw kinematics in a biomechanical size and shape space, i.e. where size is not standardised during the superimposition.^8^ This is particularly appropriate in biomechanical studies where forces, and the lever arms onto which they are applied, are central to the analysis. In the case of our analyses of motion of *A. ricciae,* the inclusion of size variation in the analysis might be pertinent where energetics of feeding is of interest. Procrustes motion analysis has also been used in invertebrates, but mainly if not exclusively in arthropods, whose joints provide well defined landmarks. For instance, it was employed to study the gait of scorpions [44] and spinneret movement in spiders [45]. As with most worm-like invertebrates, *A. ricciae* has no simple joints to landmark to track the relative positions of its body parts. However, the body of *A. ricciae* can be subdivided in a head, trunk, and foot, which are relatively easy to identify and mark up with a few landmarks. Landmarking manually frame by frame is time consuming, but doable if samples are not huge. In extensive studies of larger datasets, methods for automatic landmarking (e.g., [46]) might help by speeding up data collection and, if landmarks are easily identified by an algorithm, by increasing precision.

Simplicity is the main advantage of our approach. There are more complex alternatives, which can be considered and whose results would be interesting to compare with those of a Procrustes motion analysis. [6, 33] offer some stimulating examples. In their analyses of stereotyped behavioural sequences in *Drosophila melanogaster* Meigen, 1830, images are segmented from the background, using an algorithm that recognises the edges of the body, and the resulting data are rescaled and aligned before reducing their dimensionality using a PCA. Up to this point, the main difference with our study is that they measure the outline of the body. This clearly increases the amount of quantitative information. However, as with all methods for the analysis of outlines and surfaces, there might be subtle issues of ‘homology’ (i.e., the specific anatomical features which are mapped by the measurements [38, 39]) and the measurements only describe movements along the outline of the animal. The latter is an apparent limitation of our method too, but, if needed, one can easily add landmarks on any body part, including within the boundaries set by the body outline in an image. The next steps followed by [6, 33] take a different direction, compared to that of the present study, as they transform the PCs into a time series analysed with a Morlet wavelet transform, which creates a spectrogram to be mapped in a two-dimensional plane using t-SNE (a technique for non-linear dimensionality reduction). Finally, to detect the boundaries of behavioural regions (the equivalent of our ‘modes’), they apply a watershed transform to the t-SNE map, which is analogous to converting the image into a topographic map. The set of methods used by [6, 33] provides rich information and reduces some of the arbitrariness we discussed for our approach. Especially in organisms with complex behavioural sequences, the advantages are evident, but it might be interesting to compare results even when movement and postures are relatively simple, as in *Adineta*. A problem with tiny rotifers, however, is the requirement of long recordings of behaviour, which is relatively easy in the comparatively large fruit-fly but much harder in an animal 200 microns in length, which moves quickly out of the field of view under a microscope. With their long behavioural recordings, [33] produced evidence that, in the fruit-fly, movement is hierarchically organised, with behavioural transitions mostly occurring between specific nearby pairs of ‘postural-motion’ clusters. They also showed that there is a memory effect, which can last tens of minutes, in the transitions, which suggests internal states capable of influencing behaviours thousands of transitions away. Yet, even such a powerful information-rich approach might have some elements of arbitrariness. As in our study, the interpretation of the behavioural regions is by visual assessment of the videos and the choice of what is being measured (the outline, for instance), as well as of the algorithms to process the measurements, can affect the outcome of the analysis. The experimental settings also bias results by limiting the potential behavioural repertoire. Thus, in the present study the setting is a thin layer of water between the slide and its coverslip and in that of [6, 33] it is a featureless circular arena, where jumping or flying cannot occur. Both are simplified environments that lack external stimuli). However, in both instances, the simplified environment allows a simpler, although incomplete, range of behaviours to be rigorously recorded and described. Potentially, ‘postural-motion’ could also be mathematically modelled and simulated. With simulations, research could explore the effect of different parameters (e.g., body length or shape) and validate the predictions using experimental data (e.g., analysing feeding or motion in animals of different lengths etc.).

**Fig. 14:**
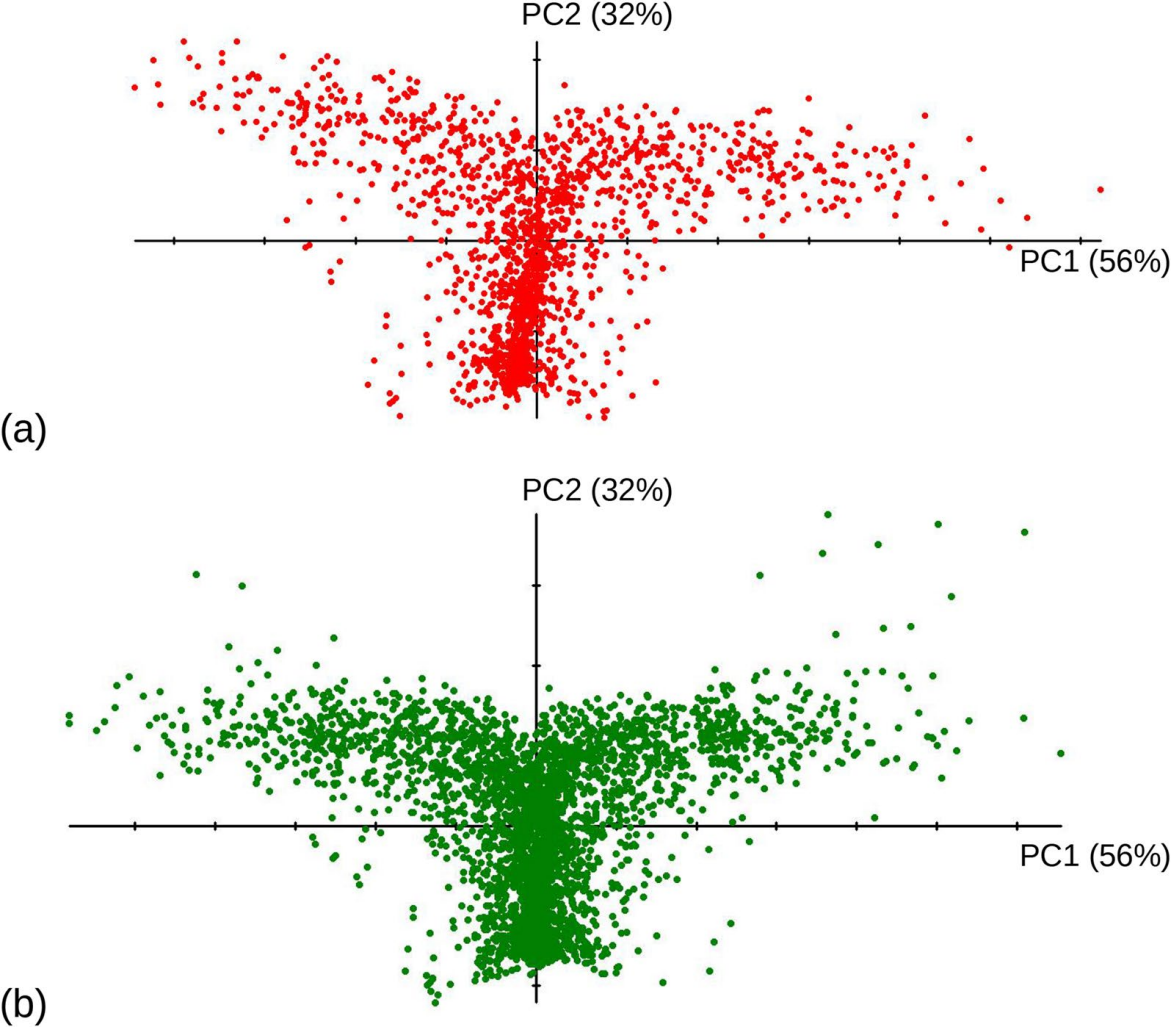
PC1-2 scatterplots from MAS (**a**–red circles) or the original total sample of 3501 frames (**b**–green circles) of feeding *Adineta ricciae*

The application of t-SNE, instead of a PCA, to summarise multivariate data is another aspect worthy of consideration in the context of Procrustes motion analysis. t-SNE [47] maximises similarity between observations (shapes, in our case) instead of maximizing total variance, as in a PCA. If the aim is to accurately depict small scale differences between observations (instead of large-scale patterns, as in a PCA) and especially when there are non-linearities, t-SNE might be a better method to obtain a low-dimensional representation of motion and detect clusters that suggest ‘modes’. Here too there are trade-offs, however. Unlike a PCA, t-SNE is sensitive to parameters and, even when parameters are fixed, employs iterations that can produce different solutions to optimise the low-dimensional representation of the data in a scatterplot. That distances are not preserved by t-SNE is a potential advantage in modelling non-linearities, but becomes more problematic using geometric morphometrics, because the geometry of the Procrustes shape space is not preserved, and the visualization of shape change along the new dimensions found in the analysis is not straightforward. For instance, one could approach the problem by finding the directions in the shape space that maximise the covariance with one or other t-SNE axis, but that is also a linear method, which inevitably entails a degree of misrepresentation of the variability summarised in a t-SNE scatterplot. In practice, in our dataset, there are non-linearities, as indicated by the Y-shaped PC1-2 scatterplots (Fig. 3, 7, 14). Yet, the vast majority of variance is captured by these two axes, so that they produce a summary of motion trajectories that can, on the one hand, be easily and effectively visualised with shape diagrams while, on the other hand, they remain accurate in terms of both the correspondence to distances in the Procrustes shape space and the representation of small to large scale relationships among frames. This second observation is supported by a simple comparison of similarity relationships using a PCA or a t-SNE. Thus, we explored the differences between PC1-2 and the bidimensional summary of t-SNE by computing matrix correlations of Euclidean distances (based on PC1-2 *vs* those using the scores of the two t-SNE axes). Using different parameters (perplexity ranging from 30 to 100) and running the analysis multiple times, the correlations varied between 0.81 and 0.83. The corresponding scatterplots confirmed the Y-shaped pattern of variation and suggested that the main differences just concern two secondary aspects. One is how the Y is rotated in the two-dimensional plots, the stem may be vertical, as in the PCA, but can also be horizontal or aligned along a diagonal. The other aspect is the relative stretching of the arms and stem of the Y. Thus, at least for a fairly simple pattern, such as that found here in *A. ricciae* during feeding, it seems that a PCA is about as accurate as a t-SNE for summarizing motion but has advantages (the preservation of Procrustes shape distances and the visualization using shape diagrams) that outweigh the effectiveness of t-SNE in describing non-linearities and small-scale differences.

### Future directions and results validation

In the Introduction we briefly anticipated a range of potential applications of the type of analysis we demonstrated in *A. ricciae*. With *A. ricciae*, for instance, it might be interesting to compare different clones or the same clone kept under different conditions. One might add food in specific spots or change food type or abundance, temperature or even check what happens if there are predators or competitors. With *A. ricciae*, the advantage is that it is easy to culture in a laboratory [10]. Laboratory conditions, however, are a simplification that may or may not be an accurate proxy for what happens in the wild. Studying motion in the field is theoretically possible at least in some organisms, but certainly it is much more challenging in terms of data collection [1]. A fascinating possibility, suggested by a colleague during a seminar (Nataša Barišić, personal communication), is that Procrustes motion analysis could be applied to also plants, whose movement may be slow but easier to record both in laboratory and in the field.

In all contexts, researchers should bear in mind the importance of accurate, representative, and large samples, as well as the need to validate results whenever possible. In this last part of the Discussion, we offer two simple examples to stress these points. Figure 14 shows PC1-2 scatterplots of Procrustes shape coordinates in *A. ricciae*: the upper plot (Fig. 14a) is the same as in Fig. 3b, which is based on the MAS of 1674 frames; the lower one is the original total sample (of which MAS is a subsample) of 3501 frames capturing feeding motion in *A. ricciae*. The pattern is very similar in both plots. However, the smaller dataset (Fig. 14a) shows a less symmetric Y, which slightly bends to the right, so that the stem deviates slightly from vertical (i.e., from PC2). In contrast, using a sample that is more than twice as large (Fig. 14b) produces a pattern that is both more symmetric and with the stem of the Y almost perfectly aligned with PC2. This small but clear difference is likely to be a consequence of the larger sampling error in the smaller, even if more homogeneous, MAS sample. With a smaller sample, accuracy is lower and ‘outliers’ (or, in fact, likely genuine cases of extreme bending during feeding) might have a more profound impact on the overall pattern suggested by the data.

Large samples are clearly important, but the external validity of the results is also crucial. Even if this is not a strong and exhaustive test, we can explore the issue in our study using the individuals and frames that we excluded from the analysis and can now be employed as a hold-out sample in relation to the main sample of 1674 frames. To explore if ‘modes’ found in MAS are generalizable and apply to the hold-out sample of 1827 frames (3,501 minus 1674), we searched for clusters of shapes in the hold-out sample using the same approach as in MAS (i.e., a k-means cluster analysis with three groups). The resulting classification represents the a priori groups (‘hold-out sample modes’) in the hold-out dataset. Then, using MAS in a discriminant analysis of shape based on its own k-means classification into the three ‘modes’ (MAS ‘training modes’, i.e., those from the cluster analysis described in the Results and summarised in Fig. 3), we derived discriminant functions for predicting ‘test modes’ in the hold-out dataset.^9^ The resulting predictions were finally compared to the ‘hold-out sample modes’. The expectation is that, if the ‘training modes’ are generalizable outside this specific sample and so the pattern found in the Procrustes motion analysis of MAS is representative for the larger laboratory population of *A. ricciae*, classification accuracy should be high. Indeed, the average validated accuracy of the MAS discriminant functions applied to the hold-out sample was 96%, which is close to perfect predictive accuracy. In fact, this degree of accuracy is more impressive if one considers that the hold-out sample was made of series of frames whose number varied among individuals and that sometimes were represented by non-consecutive motions performed in different feeding spots. Although the data are not fully independent, because, even if no frame is in common between MAS and the hold-out sample, some of the individuals are the same in both datasets, the result of the hold-out discriminant analysis is a good indication that the pattern we described in the main analysis likely holds for the entire population of *A. ricciae* from our laboratory.^10^ Whether this is also true for other populations of the same species or for different species in this genus, we cannot say but provides another interesting avenue for future research.

## Data Availability

Data are available from the Authors upon request.

## Footnotes

1 i.e., a behavioural pattern that, in spite of apparent complexity, can be numerically described by a small number of variables.

2 for a series of online videos of the movement of *Adineta* animals, consult: https://youtu.be/DI-WefcVuNQ?si=N_oDmIWMxGSDxi6O and https://www.youtube.com/watch?v=pSXlo8fuxzg

3 CS (centroid size) is the square root of the sum of squared distances between each of the six landmarks and their centroid (i.e., the average of the X and Y raw coordinates). To standardise it, the X and Y coordinates of an individual are divided by its CS.

4 The Procrustes shape distance is approximately the square root of the sum of squared distances between corresponding landmarks in two superimposed configurations. This distance measures the magnitude of the shape differences between any pair of specimens. After data are projected from the curved shape space into a tangent Euclidean space, the Procrustes shape distance can be approximated using the Euclidean distance, which corresponds to a straight line in the multivariate data space between two specimens.

5 Modified from https://math.stackexchange.com/questions/529555/signed-angle-between-2-vectors

6 Using alternative algorithms (e.g., MacQueen or LLoyd) or starting with 5, 50 or 100 sets produced identical results (not shown).

7 This ‘mode’ of motion is suggested by the observations of individuals leaving a feeding spot. The part of the videos recording this behaviour is, as explained in the Introduction, always very short, as specimens are soon lost by the operator, as they move outside the field of view of the microscope. Nonetheless, in all individuals where these, albeit short, video sequences are available, they consistently show an animal moving away in a straight line by foot telescoping.

8 Or it is restored after the superimposition [43].

9 All DAs were done using the *lda* function of *car* [48]*.*

10 The non-complete independence of the analysis and hold-out samples is in fact a minor issue. If the analysis is done using, as hold-out sample, the 20 individuals excluded from MAS (i.e., those with less than 50 frames per specimen), the validation sample is now totally independent, but the result is the same with a 96% accuracy.
